# Um mosaico histórico dos embates sobre a vacinação

**DOI:** 10.1590/S0104-59702023000100011

**Published:** 2023-04-03

**Authors:** Denise Sant'Anna

**Affiliations:** i Pontifícia Universidade Católica de São Paulo. São Paulo – SP – Brasil. dbsat@uol.com.br

O livro *Corpos inscritos: vacina e biopoder: Londres e Rio de Janeiro, 1840-1904*, da historiadora Myriam Bahia Lopes (2021), resulta de uma pesquisa pioneira sobre a vacinação antivariólica, esse “monumento da medicina científica”. Sua leitura é essencial para compreender as raízes históricas dos debates entre aqueles que são favoráveis e os que são contrários às vacinas. Mais do que isso: além de explicar posições científicas e ideológicas, recuperando a história das lutas em prol da vacinação, Myriam interroga com habilidade os interesses em jogo na construção de diversas experiências fundadoras da modernidade, tais como a formação da opinião pública, as tentativas de estabelecer um biopoder em meio às propostas de higienização e de saneamento urbanos, assim como o campo das disputas científicas fomentado pela imprensa dos dois últimos séculos. E ainda: o livro ensina o quanto as desconfianças em torno da vacinação possuem uma longa e movimentada história, tecida em meio à persistência de antigos receios diante da ameaça do contágio, mas também atravessada pela progressiva invenção de novas técnicas de imunização. Em vez de seguir uma suposta linearidade dos fatos, a autora apresenta um rico mosaico de narrativas médicas, políticas e jornalísticas, propondo hipóteses e questionamentos tão instigantes quanto essenciais para o entendimento da história do corpo e da vida urbana.

Assim, embora cada capítulo possua a sua própria “individualidade”, o conjunto da obra vai, progressivamente, complicando as certezas oriundas do senso comum: as diferenças entre os que são favoráveis e contrários à vacinação ganham complexidade ao ser investigadas à luz de seus contextos culturais, dos sentidos adquiridos pela noção de progresso e pelos interesses que motivaram cada nova descoberta científica e cada crítica publicada pela imprensa. Rio de Janeiro e Londres ocupam o centro das atenções da autora, que desenvolveu uma minuciosa pesquisa em arquivos nacionais e internacionais, contribuindo para esclarecer algumas das tentativas de construir cidades salubres e populações imunizadas. A comparação entre cidades e países revela que o debate sobre o advento da técnica da vacinação foi bem mais restrito no Brasil do que na Inglaterra. As fontes sobre os antivacinistas, mostra a autora, abundam entre os ingleses e são escassas entre os brasileiros. Os adeptos da antivacina vão sendo, a cada página, dissecados em seus significados sociais e questionados em suas convicções.

A autora conhece as armadilhas da pesquisa junto a diferentes acervos e o quanto a investigação histórica exige o conhecimento apurado das fontes analisadas e da etimologia de cada conceito. Por isso, ela perscruta os sentidos dos termos científicos e populares, além de dedicar uma parte importante do livro à análise da caricatura e do humor, justamente quando a formação da opinião pública era mobilizada tanto a favor como contra a vacina antivariólica.

O texto não apresenta uma comparação entre Inglaterra e Brasil com todos os elementos distintos e similares em relação ao tema analisado, mas oferece ao leitor um panorama geral dos significados e da história da vacinação dentro do qual a caricatura possui lugar de destaque. Nas imprensas estrangeira e brasileira, a caricatura expressou as disputas entre charlatanismo e cientificismo durante o processo de modernização da medicina, assim como algumas especificidades dos veículos de comunicação e dos interesses nacionais.

Ao longo dos capítulos, os discursos médicos, as referências à biologia, ao urbanismo e uma inspiradora análise da imprensa ilustrada concedem ao livro o poder de transportar o leitor para períodos epidêmicos distintos: primeiramente, o tempo da prática antivariólica lançada por Jenner, mais tarde, a intensificação dos estudos sobre a “verdadeira” e a “falsa” vacina – ou “vacina bastarda” – e, ainda, o movimento antivacinista das capitais portuárias de Londres e Rio de Janeiro, entre 1870 e 1904. O porto – porta e filtro – funcionando como “heterotopia do século XIX” (Foucault, 2019) é uma das hipóteses do livro, assim como a necessidade de considerar os embates sobre a vacina de modo sincrônico, respeitando as ressonâncias entre épocas e sociedades distintas. Myriam sabe o quanto imaginário e ciência não habitam planetas distintos, e, por isso, tratou a vacinação como uma espécie de vórtice em torno do qual se estrutura algo que a autora lembra ser uma “aquisição importantíssima” para o mundo contemporâneo: a vacina permite a circulação das pessoas e das mercadorias. Ou ainda: ela é, em suas palavras, “contemporânea da economia política e de suas tentativas de estabelecer as leis do mercado”.

Nada mais atual e necessário, portanto, do que abordar o tema da vacina a partir das disputas que caracterizaram a história contemporânea da imunização dos corpos e as tentativas para evitar os entraves que as epidemias colocam aos fluxos comerciais e ao desenvolvimento capitalista. Afinal, não seria justamente o problema do mercado e da circulação global de bens e pessoas um dos grandes desafios que o advento da atual pandemia provocada pelo novo coronavírus teria vindo atualizar?
